# Design of Thermal Insulation Materials with Different Geometries of Channels

**DOI:** 10.3390/polym13132217

**Published:** 2021-07-05

**Authors:** Daniela Șova, Mariana Domnica Stanciu, Sergiu Valeriu Georgescu

**Affiliations:** 1 Faculty of Mechanical Engineering, Transilvania University of Brasov, Bulevardul Eroilor 29, 500360 Brasov, Romania; sova.d@unitbv.ro; 2 Russian Academy of Natural Sciences, Sivtsev Vrazhek 29/16, 119002 Moscow, Russia; 3 Faculty of Furniture Design and Wood Engineering, Transilvania University of Brasov, Bulevardul Eroilor 29, 500360 Brasov, Romania; sergiu.georgescu@unitbv.ro

**Keywords:** thermal insulation material, porous structure, perforated extruded polystyrene, thermal conductivity

## Abstract

Investigating the large number of various materials now available, some materials scientists promoted a method of combining existing materials with geometric features. By studying natural materials, the performance of simple constituent materials is improved by manipulating their internal geometry; as such, any base material can be used by performing millimeter-scale air channels. The porous structure obtained utilizes the low thermal conductivity of the gas in the pores. At the same time, heat radiation and gas convection is hindered by the solid structure. The solution that was proposed in this research for obtaining a material with porous structure consisted in perforating extruded polystyrene (XPS) panels, as base material. Perforation was performed horizontally and at an angle of 45 degrees related to the face panel. The method is simple and cost-effective. Perforated and simple XPS panels were subjected to three different temperature regimes in order to measure the thermal conductivity. There was an increase in thermal conductivity with the increase in average temperature in all studied cases. The presence of air channels reduced the thermal conductivity of the perforated panels. The reduction was more significant at the panels with inclined channels. The differences between the thermal conductivity of simple XPS and perforated XPS panels are small, but the latter can be improved by increasing the number of channels and the air channels’ diameter. Additionally, the higher the thermal conductivity of the base material, the more significant is the presence of the channels, reducing the effective thermal conductivity. A base material with low emissivity may also reduce the thermal conductivity.

## 1. Introduction

Energy conservation by reducing heat losses, the temperature control on walls surfaces aimed for people’s protection and comfort and the increase of the operating efficiency of heating/ventilating/cooling systems, steam plants, commercial and industrial processing and supplying systems are all research directions for sustainable and intelligent development, correlated with the climate changes that have occurred in the past decade. [1,2,3,4]. The main factors influencing the heat transfer in a material are the thickness of the material and its thermal properties: thermal conductivity and specific heat. In thermal insulation, porous materials are commonly used. The porous structure uses the low thermal conductivity of the gas in the pores. At the same time, heat radiation and gas convection is hindered by the solid structure [4]. Materials with thermal insulation properties are divided according to their inner structure into:

(a) fibrous insulations, which consist in fibers with small diameter and air within the interspaces. The fibers can be in parallel or perpendicular to the surface being insulated and can be either interconnected or loose. The fibers used are silica, glass, mineral wool, slag wool and alumina silica fibers. The most used insulations of this type are glass fibers and mineral wool [5,6,7].
(b) cellular insulations that contain small individual cells, separated from each other. The cellular material can be glass or plastic foam, such as polystyrene (closed cell), polyurethane, polyisocyanurate, polyolefins or elastomers [8,9].
(c) granular insulations, which are composed of small nodules that contain voids. They are not really cellular materials, since gases can be transferred between the individual spaces. This type of insulation can be produced as bulk material or in combination with a binder and fibers in order to obtain a rigid insulation. Some examples are calcium silicate, expanded vermiculite, perlite, cellulose, diatomaceous earth and expanded polystyrene [10,11,12].

The latest research has been conducted with the aim of inventing and developing new thermal insulating materials. Jelle et al. [9] proposed innovative and robust highly thermal insulating materials. Such advanced insulation materials (AIM) are vacuum insulation materials (VIMs), gas insulation materials (GIMs), nano insulation materials (NIMs) and dynamic insulation materials (DIMs). These materials have closed pore structures (VIMs and GIMs) or either open or closed pore structures (NIMs). A vacuum insulation material (VIM) is basically a homogeneous material with a closed small pore structure filled with a vacuum with an overall thermal conductivity of less than 4 mW/(mK) in pristine condition. Maintaining the vacuum inside the pores during a long service life may be the most difficult or challenging task for the VIMs. A gas insulation material (GIM) is basically the same as a VIM, except that the vacuum inside the closed pore structure is substituted with a low-conductance gas. That is, a GIM is a homogeneous material with a closed small-pore structure filled with a low-conductance gas with an overall thermal conductivity of less than 4 mW/(mK) in pristine condition [12,13,14]. Other thermal insulation materials are vacuum insulation panels (VIPs) which represent evacuated, open-porous materials that are enveloped into a multilayer film. This is the best material in terms of thermal conductivity in pristine condition: 3–4 mW/mK. It also has a low thickness compared to traditional thermal insulation materials, i.e., polystyrene, but it has some disadvantages, such as being fragile, recording a significant decrease of performance with time and not being adaptable for construction sites without affecting the thermal conductivity. However, combining VIPs with other materials is beneficial. Among the core materials applied in VIPs are fumed silica, silica aerogels, open cell expanded polystyrene, polyurethane foams, fiberglass and composite materials. A nano-structured core material in combination with pressure reduction is favorable to be used in VIPs. The use of conventional insulation as a core material for VIPs results in the necessity of a very high quality of vacuum (~0.1 mbar). Common organic envelope materials cannot maintain this inner pressure for a long period: a rapid intake of air through the envelope will occur, resulting in a fast increase of the thermal conductivity. Solutions to maintain this high quality of pressure almost always go together with an envelope material with a higher thermal conductivity. However, VIPs are not in widespread use in buildings because of their high cost, susceptibility to perforation and the effects that worsen their performance [15,16].

Gas-filled panels (GFPs) try to minimize all parameters of heat transfer by using a low-conductive gas as the main insulator to influence both the gaseous thermal conductivity and the thermal conductivity of the solid structure. The thermal conductivity through the gas is the most important heat transfer in a GFP. As a result of the cellular structure of the GFP, the total panel has a thermal conductivity close to the still-gas thermal conductivity of the fill [16]. Some authors [6,17,18,19] proposed a simple, effective model for predicting the effective thermal conductivity of VIPs; namely, as a function of the thermal conductivity of the core materials, the equivalent thermal conductivity of the rarefied gas embraced in the core and the equivalent thermal conductivity of radiation. The micro structure of the porous core materials and vacuum degree were taken into consideration. Three VIPs were made from polyurethane foam materials, fibrous materials and nano-granular silica materials as the core materials. Surveying the excess of bulk materials now available, some materials scientists promoted a method of combining existing materials with geometric features to create multifunctional hybrid materials. By studying natural materials, the performance of simple constituent materials is improved by manipulating their internal geometry at different length scales. Craig and Grinham [6] described a method for designing building materials as heat exchangers, so that incoming fresh air can be efficiently tempered with low-grade heat while conduction losses are kept to a minimum. Any base material can be used in principle, so long as it can be manufactured with millimeter-scale air channels. Imbabi [18] introduced a new void space dynamic insulation (VSDI) technology that couples low-cost conventional insulation materials with efficient ventilation to deliver low-loss building envelopes and high indoor air quality in thin wall construction. VSDI is a new type of dynamic insulation in which the air flow is confined within a co-planar void space bounded by one or more layers of insulation material and the wall structure. The advantages of using VSDI consist in eliminating the risk of interstitial condensation and that of overheating during extreme summer months.

The research reported in this paper aims at describing a new design of building materials with improved thermal insulation properties, by applying different geometries of air channels inside the materials, thus increasing porosity and lowering the density. The method proposed is simple and cost-effective, as compared to advanced insulation materials that require the small pore structure to be filled with a vacuum or low-conductance gas and also considering the aforementioned disadvantages of these materials.

## 2. Materials, Methods and Equipment

### 2.1. Materials

**Figure 1:**
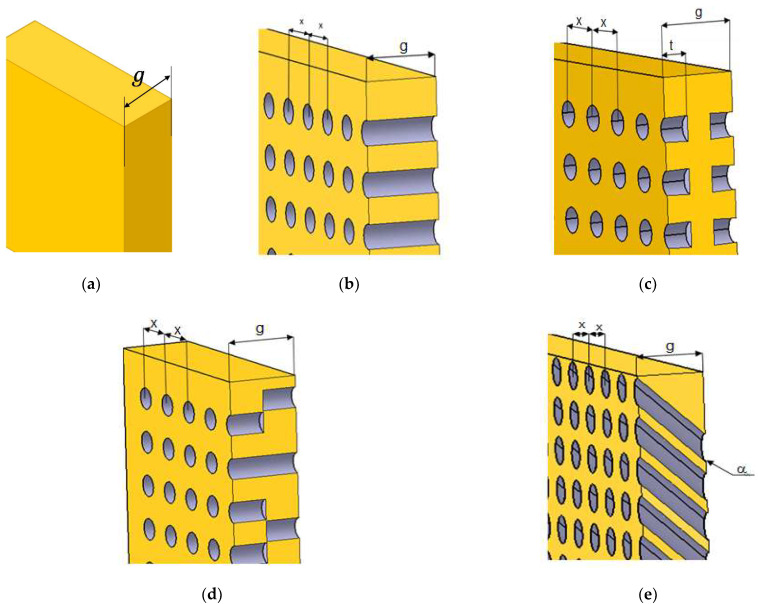
Design of thermal insulation materials with different geometries of channels: (**a**) panel without orifices, P0; (**b**) panel with perforated horizontal channels, P1; (**c**) panel with partly perforated symmetric channels, P2; (**d**) panel with partly perforated alternative channels, P3; (**e**) panel with perforated inclined channels with an angle α = 45°, P4. Legend: *g* is panel thickness; *t* is the channel length; *x* is the distance between two adjacent channels and α is the angle of inclined channels.

Starting from extruded polystyrene panels with known thermal and physical characteristics, an equidistant network (regular grids) was drawn on their surface and orifices (channels) were drilled in the nodes, either perpendicularly to the surface or inclined with a specific angle [19]. Thus, panels with horizontal or inclined channels were obtained (Figure 1). Four types of panels were designed, namely panels with perforated horizontal channels, P1 (Figure 1a), panels with partly perforated symmetric channels, P2 (Figure 1b), panels with partly perforated alternative channels, P3 (Figure 1c) and panels with perforated inclined channels with an angle α = 45°, P4 (Figure 1d). The reference panel was the extruded polystyrene (XPS) panel without orifices, P0.

The perforation was manually carried out by means of a pin on the surface of the panels, in the nodes of the grid. The thickness of the panels was *g* = 30 mm. The diameter of each orifice was *D* = 2 mm. The orifices were equally spaced at *x* = 20 mm distance from the centers (for the panels with horizontal perforation) and *x* = 30 mm for the panel with inclined channels. The distance between the center of a marginal orifice and the border of the panel was *x* as well. The lengths of the channels were *t* = 10 mm for P2 and *t* = 15 mm for P3, respectively. Table 1 indicates the physical characteristics of the panels.

### 2.2. Methods

#### 2.2.1. The Analytical Description of the Thermal Conductivity

The effective thermal conductivity was determined considering the different arrangement of the channels within the panel.

##### The Mathematical Model Applied to the Panels with Perforated Horizontal Channels

**Figure 2:**
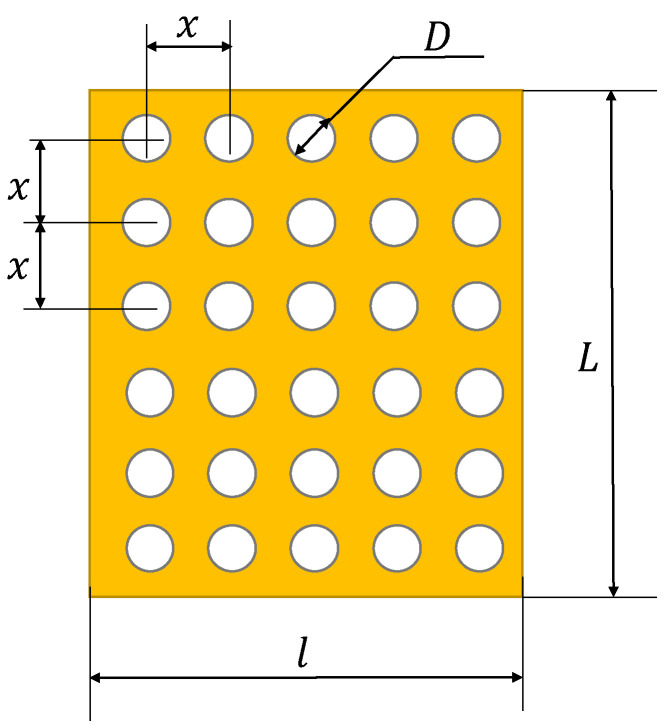
Panel with perforated horizontal orifices.

The dimensions of the panel are L×l×g, the number of channels is *n* and the diameter of each orifice, *D*. The distance between the orifices’ centers is *x*, as shown in Figure 2. The panel is quadratic, and therefore L=l.

The effective thermal conductivity was obtained by using Equations (1)–(9), considering that the resistances of XPS and air were combined into parallel circuits [8,19,20].

(1) $$\frac{1}{R_{t}}=\frac{1}{R_{XPS}}+\frac{1}{R_{air}},$$

where $R_{t}$ is the total (effective) resistance (the measurement units: K/W); $R_{XPS}$ is the resistance of polystyrene and $R_{air}$ is the resistance of air.

Equation (1) can also be written as:

(2) $$R_{t}=\frac{R_{XPS}\cdot R_{air}}{R_{XPS}+R_{air}},$$

The resistances of polystyrene and air are:

(3) $$R_{XPS}=\frac{g}{\lambda_{XPS}\cdot S_{XPS}},$$

and

(4) $$R_{air}=\frac{g}{\lambda_{air}\cdot S_{air}},$$

where *g* is the thickness of the panel (the measurement unit: m); $\lambda_{XPS}$ is the thermal conductivity of polystyrene (the measurement unit: W/mK); $S_{XPS}$ is the effective surface of polystyrene (the measurement unit: m^2^); $\lambda_{air}$ is the thermal conductivity of air (the measurement units: W/mK); $S_{air}$ is the effective surface of channels filled with air (the measurement units: m^2^).

The effective surfaces of polystyrene and channels are:

(5) $$S_{XPS}=l\cdot L-n\cdot \frac{\pi \;D^{2}}{4},$$

and

(6) $$S_{air}=n\cdot \frac{\pi \;D^{2}}{4},$$

The effective resistance can also be expressed as:

(7) $$R_{t}=\frac{g}{\lambda_{eff}\cdot S},$$

where $\lambda_{eff}$ is the effective thermal conductivity (the measurement units: W/mK); S is the surface of the panel (the measurement units: m^2^). The surface of the panel is:

(8) S=l⋅L,

By replacing Equations (3) and (4) in Equation (7), the effective thermal conductivity becomes:

(9) $$\lambda_{eff}=\frac{\lambda_{XPS}\cdot S_{XPS}+\lambda_{air}\cdot S_{air}}{S},$$

The thermal conductivity of air at the room temperature (20 °C) is $\lambda_{air}=0.024\;W/\mathrm{mK}$.

Since the channels of the panel influence the heat transfer conditions and the physical characteristics, such as density, porosity and mass, the next step is to calculate the number of channels of a quadratic panel having the arrangement of the channels indicated in Figure 2. If the number of channels on a line is $n_{{}_{L}}$, the number of channels on the entire surface of the panel becomes:

(10) $$n=n_{L}\cdot n_{L},$$

while the surface of the panel can be expressed as:

(11) $$L^{2}={\left(n_{L}+1\right)}^{2}\cdot x^{2},$$

The dimensions of the panel and the distance between channels are known quantities and thus, the number of channels on a line is:

(12) $$n_{L}=\frac{L}{x}-1,$$

The total number of channels is calculated with Equation (10):

(13) $$n={\left(\frac{L}{x}-1\right)}^{2},$$

##### The Mathematical Model Applied to the Panels with Partly Perforated Channels

**Figure 3:**
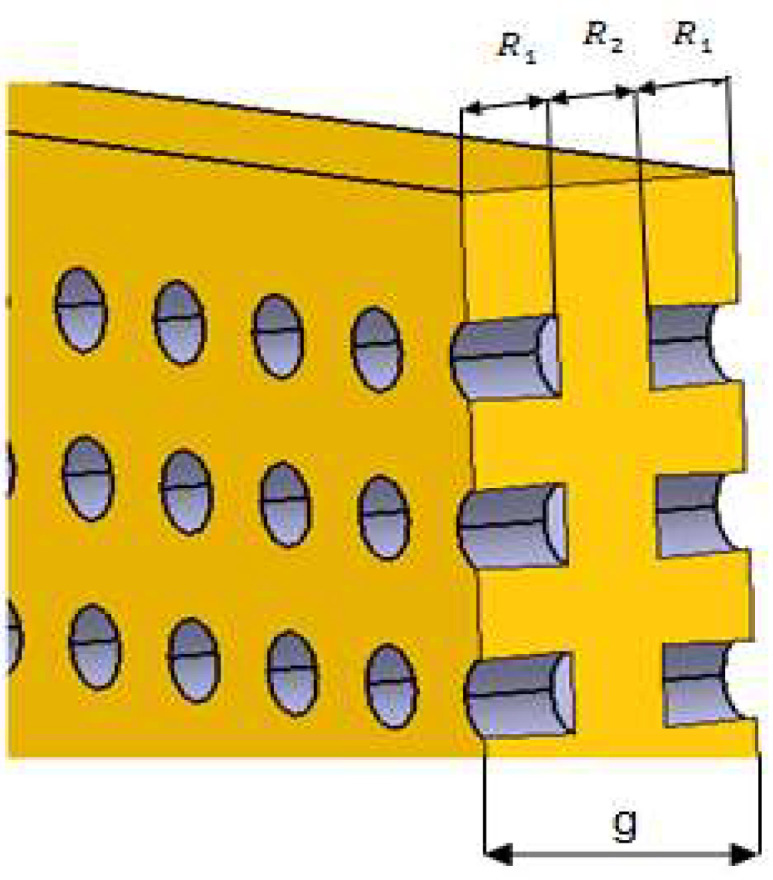
Panel with partly perforated symmetric channels.

The behavior to the heat transfer of a panel with channels having a length lower than the thickness of the panel, as indicated in Figure 3, is described by the following relations.

The resistances of XPS and air were combined into series and parallel circuits [8,9,10,11,12]. The effective resistance can be expressed according to the notations used in Figure 3 as follows:

(14) $$R_{t}=2R_{1}+R_{2},$$

where the resistance $R_{1}$ of combined polystyrene and air is:

(15) $$\frac{1}{R_{1}}=\frac{1}{R_{XPS}}+\frac{1}{R_{air}},$$

Resistance $R_{1}$ can also be written as:

(16) $$R_{1}=\frac{\frac{g}{3}}{\lambda_{eff1}\cdot S},$$

Resistance $R_{2}$ that corresponds to polystyrene is:

(17) $$R_{2}=\frac{\frac{g}{3}}{\lambda_{XPS}\cdot S},$$

The effective thermal conductivity $\lambda_{eff1}$ has a similar expression in Equation (9):

(18) $$\lambda_{eff1}=\frac{\lambda_{XPS}\cdot S_{XPS}+\lambda_{air}\cdot S_{air}}{S},$$

The effective resistance can be expressed as:

(19) $$R_{t}=\frac{g}{\lambda_{eff}\cdot S},$$

From Equations (14), (16)–(19), the effective thermal conductivity becomes:

(20) $$\lambda_{eff}=\frac{\lambda_{eff1}\cdot \lambda_{XPS}}{\frac{2}{3}\lambda_{XPS}+\frac{1}{3}\lambda_{eff1}},$$

or

(21) $$\lambda_{eff}=\frac{3\left(\lambda_{air}\cdot \lambda_{XPS}\cdot S_{air}+\lambda_{XPS}{}^{2}\cdot S_{XPS}\right)}{2\lambda_{XPS}\cdot S+\lambda_{air}\cdot S_{air}+\lambda_{XPS}\cdot S_{XPS}},$$

##### The Mathematical Model Applied to the Panels with Perforated Inclined Channels with an Angle α

**Figure 4:**
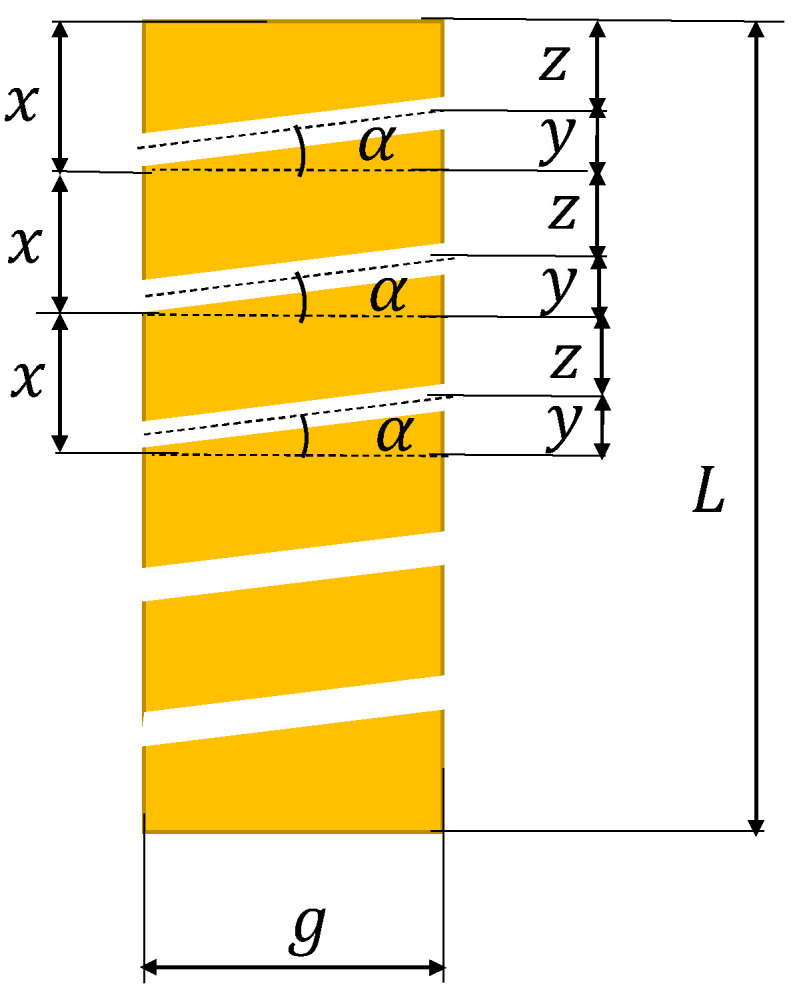
Panel with perforated inclined channels (*g* panel thickness, *x* distance between the centers of channels, α inclination angle).

In this case, the model is developed for the determination of the inclination angle, which depends on the thickness of the panel and the distance between the centers of the channels on both panel surfaces (Figure 4).

The following equations describe the relation between the dimensional quantities:

(22) x=y+z,    y≤x,

and

(23) x≥g⋅tgα,

Thus:

(24) x≥g⋅tgα,

If the distance *x* is selected, the angle can be found from:

(25) $$tg\alpha \leq \frac{x}{g},$$

If the distance between the centers of the channels increases, the number of channels decreases and if the inclination angle decreases, dimensions *z* and *x* decrease. The thermal conductivity is determined using the same mathematical model that was applied to the panels with perforated horizontal channels [9].

According to the aforementioned equations, the influence of the different variables (thickness, number of channels, magnitude of the diameter, type of channel–perforated, partly perforated or inclined at different angles, etc.) on the thermal conductivity can be analyzed [21,22]. The calculation methods are valid as long as the characteristics are uniform and the material inhomogeneity and technological errors in performing perforations are neglected (variable diameter, the inclination angle is not maintained constant, the material breaks on the opposite surface to the perforation side, the distance between the centers of the channels is variable, the panel is locally pressed, etc.). Therefore, the results of the analytical methods need experimental validation.

#### 2.2.2. Experimental Setup

The control panel and innovative panels used had overall dimensions of 0.6 m in length, 0.6 m in width and 0.03 m in thickness, which correspond to the dimensions required by the heat flow meter (Netzsch HFM 436/6 Lambda—NETZSCH-Gerätebau GmbH, Selb, Germany), and were subjected to three temperature regimes in order to measure the thermal conductivity of each panel. The heat flow meter measurement method was based on the European standards EN 12667 and EN 12939 [23,24].

The measurements consisted in setting the panel between two plates with different temperatures. The rate of heat flow per unit surface was measured by means of heat flux transducers. The electrical signal generated by a transducer is proportional to the heat rate applied to the surface of the sensor. The magnitude of the rate of heat flow depends on different factors; namely, thermal conductivity, panel thickness, temperature difference, area of the panel surface. Fourier’s law was applied to express the rate of heat flow.

Experiments were carried out for three temperature regimes, as indicated in Table 2.

**Figure 5:**
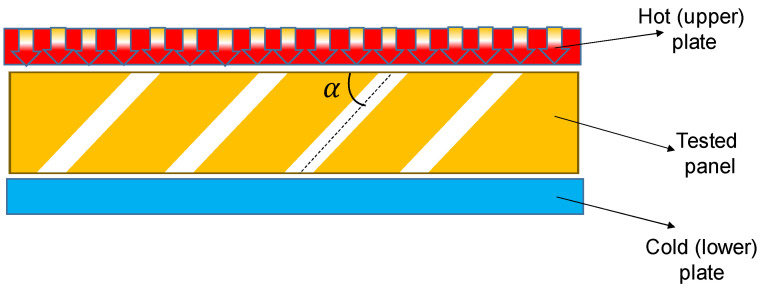
Orientation of the panel with inclined channels in the heat flow meter (P4—panel with perforated inclined channels with an angle α = 45°).

Figure 5 shows the orientation of the panel with inclined channels in the heat flow meter.

## 3. Results and Discussion

### 3.1. Analytical Models

The effective thermal conductivity ($\lambda_{eff}$) of porous materials can be calculated by using the thermal conductivity of the solid ($\lambda_{s}$), the thermal conductivity of the air within the pores ($\lambda_{air}$), the number of pores (*n*) and the diameter of a pore (*D*):

(26) $$\lambda_{eff}=\frac{\lambda_{s}\cdot (l\cdot L-n\frac{\pi D^{2}}{4})+\lambda_{air}\cdot n\frac{\pi D^{2}}{4}}{l\cdot L},$$

Equation (26), which is similar to Equation (9), was derived from the steady, one-dimensional heat transfers by pure conduction in a multilayer panel, considering the heat flux perpendicular to the panel surface. The diameter of an orifice is limited to a few millimeters in order that air flow inside channels due to temperature gradients is reduced, and therefore it can be neglected. The assumption is made that the thermal conductivity of air depends on the average panel temperature. Equation (26) is similar to that indicated by Kan et al. [16] for VIPs, where the effective thermal conductivity of a porous medium is expressed as a function of its porosity (ξ), thermal conductivity of the solid matrix ($\lambda_{s}$), thermal conductivity of the rarefied gas ($\lambda_{g}$), and thermal conductivity of radiation ($\lambda_{r}$), as follows:

(27) $$\lambda_{eff}=\left(1-\xi \right)\lambda_{s}+\xi \lambda_{g}+\lambda_{r},$$

They also ignored the thermal convection between the solid wall and the filled gas, due to the low pressure of air at normal temperature, but they considered the effect of thermal radiation inside pores.

The equation of the thermal conductivity accounting for the radiation transfer between internal channel surfaces of the panel is indicated by the same authors [7] as follows:

(28) $$\lambda_{r}=4l_{c}\sigma \left(T_{1}+T_{2}\right)\left(T_{1}^{2}+T_{2}^{2}\right)/3\varphi ,$$

where, $l_{c}$ is the thickness of the core material, φ is the attenuation coefficient for porous media (φ=445), σ is Boltzmann constant ($\sigma =5.6697\times 10^{-8}\;W/m^{2}K^{4})$. Jelle et al. [9,25] applied the Stefan–Boltzmann equation to find the total radiation heat flux through a material with n air gaps in series with infinite parallel surfaces of equal emissivity, which may be approximated as n pores along a given horizontal line in the material. Accordingly, the radiation thermal conductivity ${}_{r}$ in the nano insulation materials’ pores may be approximately calculated by:

(29) $$\lambda_{r}=\frac{\sigma d\left(T_{1}^{4}-T_{2}^{4}\right)}{\left(\frac{2}{\varepsilon }-1\right)\left(T_{1}-T_{2}\right)},$$

where d is the pore diameter, ε is the emissivity of inner pore walls. The emissivity of polystyrene is 0.9 according to [8].

**Figure 6:**
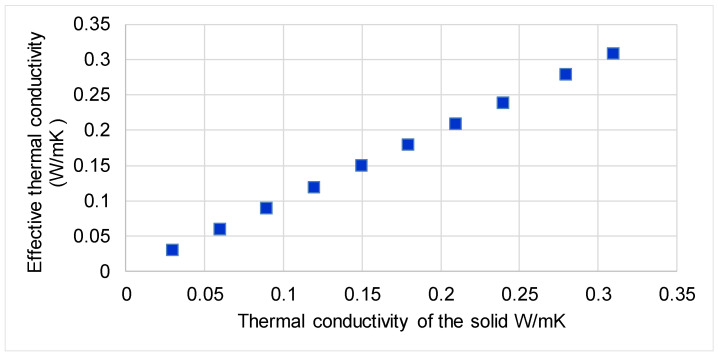
Variation of calculated effective thermal conductivity of the perforated panel as a function of the thermal conductivity of the solid.

It was observed from the analytical modeling of the panels with perforated horizontal channels that the increase of the thermal conductivity of the solid determined the linear increase of the effective thermal conductivity (Figure 6).

**Figure 7:**
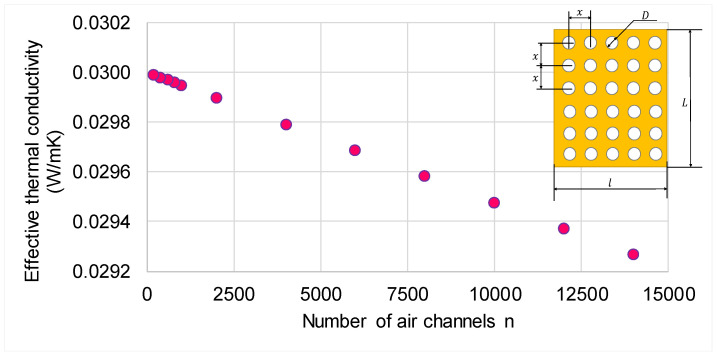
Variation of calculated effective thermal conductivity of the perforated panel as a function of the number of channels.

Therefore, the higher the thermal conductivity of the solid, the more significant is the presence of the channels, thus reducing the effective thermal conductivity of the panel. If the number of channels increases, the effective thermal conductivity decreases, as shown in Figure 7. For example, increasing the number of channels 70 times, the effective thermal conductivity reduces by 2.46%.

**Figure 8:**
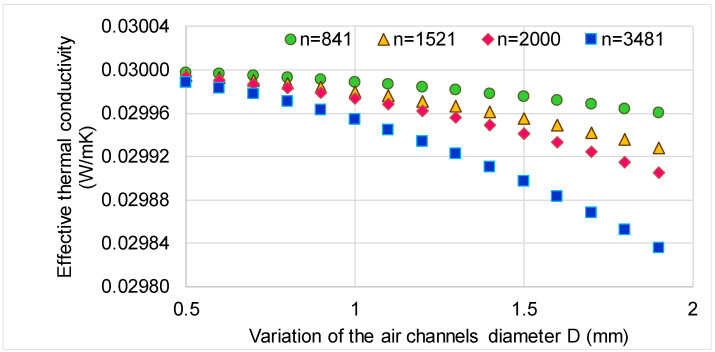
Variation of calculated effective thermal conductivity of the perforated panel as a function of the diameter of the channels. Legend: *n*—number of channels.

In order to analyze the influence of the number of channels and the diameter of the channels on the effective thermal conductivity of the panel, different cases were analyzed (Figure 8). It can be observed from Figure 8 that the effective thermal conductivity decreases when the number of channels increases 4 times and the diameter increases 3.8 times, since the density is reduced and the porosity is increased. However, it is not indicated to increase the diameter too much because air flows may occur within the channels due to temperature gradients, which make possible a heat transfer by convection. A study of optimum channel diameter may be performed in future research.

### 3.2. Experimental Results

**Figure 9:**
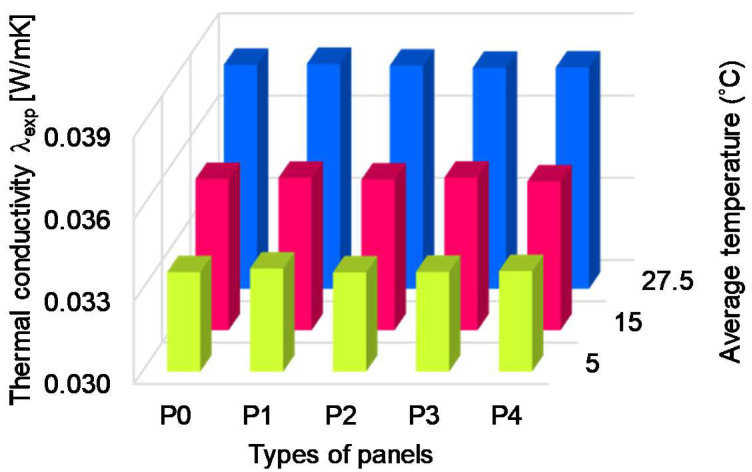
Variation of thermal conductivity of panels obtained from experiments as a function of the channels’ arrangement and temperature regime.

In all cases of tests the thermal conductivity decreased with an increasing temperature difference (Table 3). It can be observed that for the first temperature regime, the thermal conductivity of the panels with partly perforated symmetric channels decreased as compared to the XPS panel. Some condensation may have occurred in the air channels because of the negative temperature of the cold plate of the experimental device, thus improving the heat conduction. For the second temperature regime, the thermal conductivity of the partly perforated symmetric XPS panel and the panel with inclined channels slightly decreased as compared to the simple XPS panel. The decrease was higher for the XPS panel with inclined channels. As for the third temperature regime, the thermal conductivity of all perforated XPS panels decreased, except that of the panel with horizontal perforated channels. The increase of the thermal conductivity of the panel with horizontal channels is explained by the parallel arrangement of the solid and air, as resistive elements, which enhanced the heat transfer. The decrease was higher for the XPS panels with partly perforated alternative channels and inclined air channels. Figure 9 indicates the variation of the thermal conductivity obtained from experiments considering the type of the panel and the temperature regime.

The thermal conductivity depends on the average temperature of the panel. The average temperatures were 5 °C (first regime), 15 °C (second regime) and 27.5 °C (third regime), respectively, as can be seen from Table 3. Accordingly, the thermal conductivity increased with the increase of the average temperature. When the temperature exceeded 20 °C on both faces of the panel (third regime), the insulation capacity decreased by 13% as compared to the first regime.

The differences between the thermal conductivity of simple XPS and perforated XPS panels were small, but the latter could be improved by increasing the number of channels and air channels diameter, i.e., increasing the porosity. The mean free path of the air molecules should be larger than the pore diameter, thus hindering collisions between molecules and decreasing thermal conductivity.

**Figure 10:**
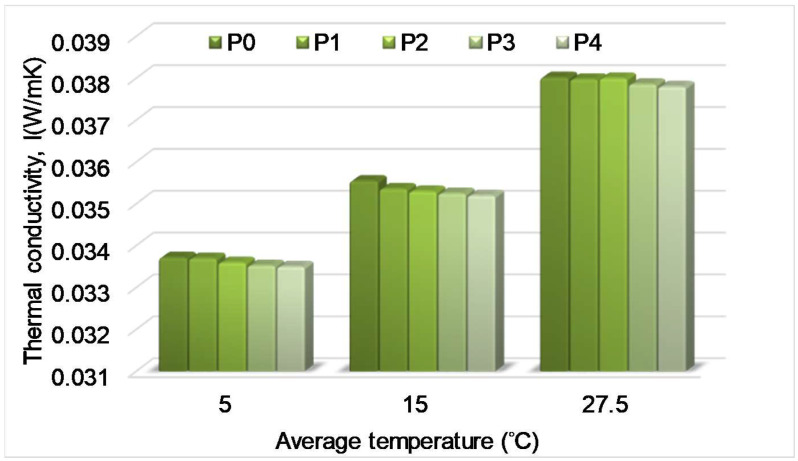
Variation of thermal conductivity of panels obtained for different types of panels in experimental conditions if neglecting the radiation heat transfer. Legend: P0—XPS control panel; P1—panel with perforated horizontal channels; P2—panel with partly perforated symmetric channels; P3—panel with partly perforated alternative channels; P4—panel with perforated inclined channels with an angle α = 45°.

The experimental values include the effect of the radiation heat transfer inside air channels. If neglecting this component, a further reduction of the thermal conductivity may be achieved (Figure 10), which is the lowest limit of the thermal conductivity that can be achieved. 

In this case, the thermal conductivity of the panel P4 with inclined orifices decreased by 1.2, 2.2 and 1.8%, respectively for the three temperature regimes. The radiation thermal conductivity depended on the dimensions of the pores or panel and emissivity of the base material. Lowering dimensions and using a base material with low emissivity would decrease the radiation thermal conductivity. Additionally, a base material with high thermal conductivity is influenced by the presence of air channels inside the panel, decreasing significantly its thermal conductivity.

## 4. Conclusions

A new design of existing building materials by generating air channels inside was reported in the paper. XPS panels were perforated horizontally or at an angle of 45°. The presence of air channels reduced the thermal conductivity, but the reduction was more significant in the panels with inclined channels, P4.

An optimized design would assume the reduction of the channel diameter and the increase in the number of channels. A base material with low emissivity may also reduce the thermal conductivity.

Perforated panels can be included in a multilayer insulation structure. The future work will continue with implementation of different thicknesses and a sandwich structure of panels.

## Figures and Tables

**Table 1:** The physical features of panels.

Code of Panel	Mass m (kg)	Density ρ (kg/m^3^)	Dimensions		
			Length (m)	Width (m)	Thickness (m)
P_0_	0.311	28.79	0.6	0.6	0.03
P_1_	0.302	27.96	0.6	0.6	0.03
P_2_	0.308	28.51	0.6	0.6	0.03
P_3_	0.303	28.05	0.6	0.6	0.03
P_4_	0.300	27.77	0.6	0.6	0.03

**Table 2:** The input data of HFM controller.

Hot Plate Temperature (°C)	Cold Plate Temperature (°C)	Average Temperature (°C)	The Absolute Value of the Temperature Difference between the Two Control Thermocouples (°C)
−10	20	5	30
10	20	15	10
35	20	27.5	15

**Table 3:** Thermal conductivity of panels tested at three temperature regimes.

Average Temperature (°C)	The Absolute Value of the Temperature Difference Δt (°C)	$\mathrm{Thermal}\;\mathrm{Conductivity}\;\lambda_{exp}\;[W/\mathrm{mK}]$				
		P_0_	P_1_	P_2_	P_3_	P_4_
5	30	0.03363	0.03376	0.03362	0.03364	0.03367
15	10	0.03552	0.03556	0.03550	0.03557	0.03543
27.5	15	0.03818	0.03821	0.03815	0.03807	0.03808

## Data Availability

Not applicable.
